# Full length genomic sanger sequencing and phylogenetic analysis of Severe Acute Respiratory Syndrome Coronavirus 2 (SARS-CoV-2) in Nigeria

**DOI:** 10.1371/journal.pone.0243271

**Published:** 2021-01-11

**Authors:** Joseph Ojonugwa Shaibu, Chika K. Onwuamah, Ayorinde Babatunde James, Azuka Patrick Okwuraiwe, Olufemi Samuel Amoo, Olumuyiwa B. Salu, Fehintola A. Ige, Gideon Liboro, Ebenezer Odewale, Leona Chika Okoli, Rahaman A. Ahmed, Dominic Achanya, Adesegun Adesesan, Oyewunmi Abosede Amuda, Judith Sokei, Bola A. O. Oyefolu, Babatunde Lawal Salako, Sunday Aremu Omilabu, Rosemary Ajuma Audu

**Affiliations:** 1 Microbiology Department, Centre for Human Virology and Genomics, Nigerian Institute of Medical Research, Lagos, Nigeria; 2 Biochemistry Department, Nigerian Institute of Medical Research, Lagos, Nigeria; 3 Department of Medical Microbiology and Parasitology, Centre for Human and Zoonotic Virology, College of Medicine, Lagos University Teaching Hospital, Lagos, Nigeria; 4 Department of Cell Biology and Genetics, University of Lagos, Akoka, Lagos, Nigeria; 5 Centre for Tuberculosis Research, Nigerian Institute of Medical Research, Lagos, Nigeria; 6 Department of Microbiology, Virology Research Group, Lagos State University, Ojo, Lagos, Nigeria; 7 Nigerian Institute of Medical Research, Yaba, Lagos, Nigeria; "INSERM", FRANCE

## Abstract

In an outbreak, effective detection of the aetiological agent(s) involved using molecular techniques is key to efficient diagnosis, early prevention and management of the spread. However, sequencing is necessary for mutation monitoring and tracking of clusters of transmission, development of diagnostics and for vaccines and drug development. Many sequencing methods are fast evolving to reduce test turn-around-time and to increase through-put compared to Sanger sequencing method; however, Sanger sequencing remains the gold standard for clinical research sequencing with its 99.99% accuracy This study sought to generate sequence data of SARS-CoV-2 using Sanger sequencing method and to characterize them for possible site(s) of mutations. About 30 pairs of primers were designed, synthesized, and optimized using endpoint PCR to generate amplicons for the full length of the virus. Cycle sequencing using BigDye Terminator v.3.1 and capillary gel electrophoresis on ABI 3130xl genetic analyser were performed according to the manufacturers’ instructions. The sequence data generated were assembled and analysed for variations using DNASTAR Lasergene 17 SeqMan Ultra. Total length of 29,760bp of SARS-CoV-2 was assembled from the sample analysed and deposited in GenBank with accession number: MT576584. Blast result of the sequence assembly shows a 99.97% identity with the reference sequence. Variations were noticed at positions: nt201, nt2997, nt14368, nt16535, nt20334, and nt28841-28843, which caused amino acid alterations at the S (aa614) and N (aa203-204) regions. The mutations observed at S and N-gene in this study may be indicative of a gradual changes in the genetic coding of the virus hence, the need for active surveillance of the viral genome.

## Introduction

It is no longer news that one of the major battle humanity is fighting currently is the fight against SARS-CoV-2. It started in Wuhan, China in December 2019 **[1]** but has spread to most countries in the world. As the global community battles this novel virus through aggressive screening of individuals for the presence or absence of infection, there is also a salient need for a genetic characterization which will help in the long-term management of the infection. A pool of sequences from various countries of the world would help in assessing the mutational tendencies of the virus as it crosses different geographical conditions and people of different cultures, genetic variations and immune status **[2].** Complete genome sequencing of viruses is an essential tool for the development of diagnostics and vaccines, studying virus pathogenicity and virulence, tracking evolutionary paths and studying the genetic association between viruses and their hosts **[3]**. Optimized sets of primers for Sanger sequencing could be of help for whole genome generation especially for laboratories who lack Next Generation Sequencing platform but have Genetic analyzer. This method though an older methodology in genome sequencing, with its 99.99% accuracy, remains a ‘gold standard’ for clinical research sequencing (*https://www.thermofisher.com/blog/behindthebench/when-do-i-use-sanger-sequencing-vs-ngs-seq-it-out-7/*). A critical determinant for successful full genome Sanger sequencing of any pathogen is the availability of primers that target only the pathogen of interest reliably but not the sequences of host genes or other pathogens **[4]**.

The rapid spread of SARS-CoV-2 across countries calls to questions on whether its evolution is mutation driven. It is reported that genomes with new variations are emerging as the virus moves across diverse environmental conditions **[5]**. Hence, there is a need for mutation monitoring of the virus. A study reviewed that RNA viruses’ mutation rate is higher than that of their hosts and this rate plays an important role in virulence modulation and in the development of traits that are useful for viral adaptation **[6]**. Also, variations have been noticed at ORF1ab, S, ORF3a, ORF8 and N regions, in which nt28144 in ORF8 and nt8782 in ORF1a showed variation rates of 30.53% and 29.47%, respectively **[7]**. Variations at the nucleotide level which is proposed to be one of the most significant measures of viral evolvement, also contributes to the adaptation of the virus in every condition it finds itself thus creating a balance between its genetic information and genome variability **[8,9]**.

SARS-CoV-2 is a positive-sense single-stranded RNA virus. Studies showed that it has a zoonotic origin with genetic similarity to bat coronaviruses **[10,11]**. The whole genome size of the SARS-CoV-2 varies from 29800bp to 30000 bp and it consists of a long open reading frame (ORF1ab) of about 21290bp encoding orf1ab polyproteins, structural proteins such as surface (S) about 3822bp, envelope (E) about 228bp, membrane (M) about 669bp, and nucleoprotein (N) about 908bp. It also has additional 6 accessory proteins, encoded by ORF3a, ORF6, ORF7a, ORF7b, and ORF8 genes **[12]**.

This study explored the use of Sanger technology to sequence and characterize the whole genome of SARS-CoV-2 using specific overlapping primers designed from the various genes of the virus.

## Materials and methods

### Ethics

Ethical approval (IRB/19/025) was obtained from Nigerian Institute of Medical Research Internal Review Board for the collection of samples for this study. Informed consents were filled by participants who agreed for their samples to be taken for diagnosis and research.

### Samples

In April 2020, fifty (50) nasopharyngeal swabs were collected from suspected cases at the modified COVID-19 drive-through testing facility at the Nigerian Institute of Medical Research (NIMR), Yaba, Lagos. The samples were collected into a Viral Transport Medium (VTM) taken to a Biosafety level III laboratory within the Institute for inactivation by aliquoting into lysis buffer and leaving to stand at room temperature for 10 minutes. Thereafter, the inactivated aliquots were moved to a BSL II laboratory for RNA extraction and sequencing. The sample used for this study coded 57752 in our database was selected for Sanger whole genome sequencing based on its relatively high Ct value when compared to others.

#### RNA extraction

The viral nucleic acid from the inactivated samples aliquots were extracted using the QIamp RNA extraction kit (Qiagen, Maryland, United States) according to the manufacturer’s instructions.

#### RT-qPCR

One-step reverse transcriptase (RT) real-time (qPCR) was carried out to detect SARS-CoV-2 using qPCR assay designed by BGI, Shenzhen, China. The process contained 18.5μl of nucleic acid mix, 1.5μl of enzyme mix and 10μl of RNA in a total reaction volume of 30μl. RT-qPCR cycling was performed on QuantStudio 3 (Applied Biosystems) as follows: 50°C for 20 minutes, 95°C for 10 minutes, then 40 cycles of 15s at 95°C and 30s at 60°C.

### Primers

Specific overlapping primers for the amplification of the entire length of the virus were designed in-house from conserved regions using Oligo Primer Analysis Software v.7 to obtain amplicons of 1300-1600bp. These primers were synthesized by Macrogen Europe B.V., Amsterdam, Netherlands.

### Nucleic acid amplification

Synthesis of highly structured and long cDNA fragments was performed using SCRIPT cDNA Synthesis Kit (Jena Bioscience, GmbH, Germany) according to the manufacturer’s instruction. Gradient PCR was carried out using the primers pairs, to optimize the annealing temperature and amplification of targeted fragments using MiniAmp Plus Thermal Cycler (Applied Biosystems). The PCR process was performed in a total volume of 50μl comprising 10μl of 5x Red Load Taq Master Mix, 5ul each of 10μM forward and reverse primers, 3ul of cDNA template and PCR grade water to make up the volume. PCR cycling conditions: 1 cycle of 94°C for 2 minutes; 30 cycles of 94°C for 30 secs, 53–64°C for 30secs, 72°C for 2 minutes and 1 cycle of 72°C for 2 minutes were used. The amplicons were analysed by 1.8% agarose gel electrophoresis.

### Cycle sequencing and ABI analysis

In this study, we used Sanger technology to sequence and characterize the whole genome of SARS-CoV-2 using specific overlapping primers designed from the various genes of the virus.

The amplicons were purified using HT ExoSAP-IT (Thermo Fischer scientific) according to the manufacturer’s instructions. The purified amplicons were quantified using Qubit 4 Fluorimeter (Thermo Fischer Scientific, CA). Sequencing was performed using the BigDye Terminator kit v. 3.1 and cleaned up with BigDye XTerminator v. 3.1 (Applied Biosystems, Foster City, CA). The purified products of the cycle sequencing were analysed on the ABI 3130xl Genetic Analyser (Applied Biosystems). This process was carried out at the Centre for Human Virology and Genomics, NIMR. Sequences generated were assembled using DNASTAR Lasergene 17 SeqMan Ultra in alignment with the reference genome (NC_045512.2).

### Phylogenetic analysis

Thirty four (34) whole genome sequences of Sars-CoV-2 were downloaded from GenBank of NCBI (https://www.ncbi.nlm.nih.gov/GenBank) using the blastn search tool with SARS-CoV-2 as query. The sequences were aligned alongside the sequence data generated from this study using MAFFT v. 7 **[13]** and BioEdit software. Phylogenetic analysis was done using the MEGA X **[14]**, applying the Maximum likelihood method (ML) andTamura-Nei model **[15]**. The bootstrap consensus tree inferred from 1000 replicates based on General Time Reversible (GTR) substitution model with gamma distribution.

## Results and discussion

### PCR Virus detection and amplification

A Ct-value of 19 was observed on QuantStudio 3 (QS-3) qPCR system for sample NGN57752 using SARS-CoV-2 real time PCR assay developed by BGI, China. These Ct value was less than the cut-off Ct value of 38 as set by the assay. Hence, this result shows the presence of the virus in the sample assayed. The cDNA synthesized from the RNA according to the process described above gave a yield of 4.08 ng/μl. The amplicons from PCR process were loaded on 1.8% agarose gel and ran on CyFox^®^ live imaging and gel electrophoresis device (Sysmex Partec, GmbH).

**Fig 1:**
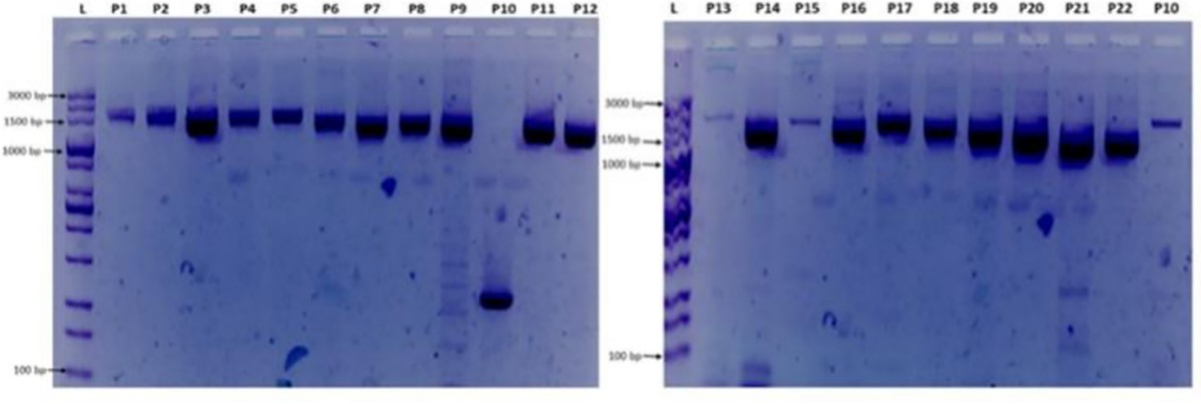
Gel electrophoresis of primer combinations (P1-P22) used for amplification and sequencing. L = 100 bp ladder, P1 = NIMR_CoV1, P2 = NIMR_CoV2, P3 = NIMR_CoV3, P4 = NIMR_CoV4, P5 = NIMR_CoV5, P6 = NIMR_CoV6, P7 = NIMR_CoV7, P8 = NIMR_CoV8, P9 = NIMR_CoV9, P10 = NIMR_CoV10, P11 = NIMR_CoV11, P12 = NIMR_CoV12, P13 = NIMR_CoV13, P14 = NIMR_CoV14, P15 = NIMR_CoV15, P16 = NIMR_CoV16, P17 = NIMR_CoV17, P18 = NIMR_CoV18, P19 = NIMR_CoV19, P20 = NIMR_CoV20, P21 = NIMR_CoV21, P22 = NIMR_CoV22. Note: P10 on the first gel image (Fig 1 above) with band size of about 350bp was not used in the sequencing process; instead, the P10 on the second gel was used. The gel images were obtained from sample NGN57752 with accession number: MT576584.1.

The primers developed and successfully used in this study to generate a whole genome will enable laboratories with access to only Sanger technology, to generate sequence data for their countries. This could increase representation of SARS-CoV-2 genome data from more countries to available genome data in various data banks across the world. The Sanger method, an older technology when compared with NGS, has a lower throughput, is more expensive if you want to use it for several samples and pathogens with large genome length. Also, the quality of Sanger sequences is often not very good at the extremes due to the fact that those are the sites where the primers bind before extension. However, with high quality read lengths of between 500-900bp per primer, it tends to minimize errors during assembly when compared to NGS reads. The primers used in this studies are able to amplify amplicons from RNA with Ct values of ≤ 30 in first round amplification. Fig 1 below shows gel images from the first round amplification using the primers in Table 1 above.

**Table 1:** Amplification and sequencing primers.

Primer Name	Region of amplification	Primer orientation	Oligo sequence	Length coverage	Optimized Annealing temperature (˚C)
NIMR_CoV1	ORF1ab/5' NCR	Forward	AACAAACCAACCAACTTTCG	26–1491	55
		Reverse 1	CAACCAACATAAGAGAACACAC		
		Reverse 2	CAGCCTGCAGAAGATAGACG		
NIMR_CoV2	ORF1ab	Forward	TGTCCAGCATGTCACAATTCAG	1373–2944	57
		Reverse	CCACTCATCTAAATCAATGCCC		
NIMR_CoV3		Forward 1	GGTACAGAAGTAAATGAGTTCGCC	2858–4505	60
		Reverse 1	TCTAGCACCATAATCAACCACACC		
		Forward 2	CACTGTAGAGGAGGCAAAGAC		
		Reverse 2	AAGCTTGCGTTTGGATATGGTTGG		
NIMR_CoV4		Forward	CCTGTCTGTGTGGAAACTAAAGCC	4433–6016	58
		Reverse	AAGCTTGCGTTTGGATATGGTTGG		
NIMR_CoV5		Forward 1	CAGAATACAAAGGTCCTATTACGG	5835–7391	57
		Reverse 1	CATTCTAACCATAGCTGAAATCGG		
		Forward 2	TCACTATTGCAACCTACTGTACTGG		
		Reverse 2	GCTAAACCATGTGTCAAAATCAGC		
NIMR_CoV6		Forward	TTTCTATGTACTTGGATTGGCTGC	7276–8759	57
		Reverse	GCTAAACCATGTGTCAAAATCAGC		
NIMR_CoV7		Forward	TAACAACAAAGATAGCACTTAAGG	8526–9973	57
		Reverse	AGTTACTGAAGTCATTGAGAGCC		
NIMR_CoV8		Forward 1	TGATGTGCTATTACCTCTTACGC	9850–11299	59
		Reverse 1	TGCATACATAACACAGTCTTTTAGC		
		Forward 2	ATGCCTTTTTACCTTTTGCTATGGG		
		Reverse 2	ACAGCAGAATTGGCCCTTAAAGC		
NIMR_CoV9		Forward	TTGTTACCTTCTCTTGCCACTGTAG	11183–12653	57
		Reverse	ACAGCAGAATTGGCCCTTAAAGC		
NIMR_CoV10		Forward	CATAATACCTCTTACAACAGCAGCC	12445–14035	57
		Reverse	TGTCAGTACACCAACAATACCAGC		
NIMR_CoV11		Forward	CTAAATACACAATGGCAGACCTCG	13799–15299	55
		Reverse	TAGGCATGGCTCTATCACATTTAG		
NIMR_CoV12		Forward	AGCTGGTGTCTCTATCTGTAGTAC	15111–16577	58
		Reverse	ATGTAATCACCAGCATTTGTCC		
NIMR_CoV13		Forward	CCCATTAGTTTTCCATTGTGTGC	16468–17968	57
		Reverse	GCAACTTGTCATAAAGGTCTCTATC		
NIMR_CoV14		Forward	CATCACAGGGCTCAGAATATGAC	17840–19280	58
		Reverse	TACATACAAACTGCCACCATCAC		
NIMR_CoV15		Forward	GGAAGTTCTATGATGCACAGCC	19082–20476	57
		Reverse	AGAACACACACACTTAGATGAACC		
NIMR_CoV16		Forward	TTTTAGTCATAGTCAGTTAGGTGG	20337–21827	61
		Reverse	CTCAGTGGAAGCAAAATAAACACC		
NIMR_CoV17	S-gene	Forward 1	AACCAGAACTCAATTACCCCCTGC	21619–23266	57
		Reverse 1	CAGCATCAGTAGTGTCAGCAATG		
		Forward 2	ATCTCTGCTTTACTAATGTCTATGC		
		Reverse 2	TGGTTAGAAGTATTTGTTCCTGG		
NIMR_CoV18		Forward 1	TCAATGGTTTAACAGGCACAGG	23190–24698	57
		Reverse 1	GGGAAGGACATAAGATGATAGCC		
		Forward 2	CTATGAGAACCAAAAATTGATTGCC		
		Forward 2	TGGACATGTTCTTCAGGCTCATC		
NIMR_CoV19	ORF3a	Forward	AGGCTGAAGTGCAAATTGATAGG	24525–26104	57
		Reverse	TGGACATGTTCTTCAGGCTCATC		
NIMR_CoV20	E-gene	Forward 1	CGCTCTCACTCAACATGGCAAGG	25971–27517	57
		Reverse 1	CTCTTCCATATAGGCAGCTCTCCC		
		Forward 2	GGAATCTGGAGTAAAAGACTGTG		
		Reverse 2	GAGGATGAAATGGTGAATTGCC		
NIMR_CoV21	M	Forward	TCTTGGCACTGATAACACTCGC	27410–28682	57
		Reverse	ATCTTTTGGTGTATTCAAGGCTCC		
NIMR_CoV22	N-gene, ORF6,7 8&10	Forward 1	TGTCACTAAGAAATCTGCTGC	28435–29777	57
		Forward 2	CGATATATAGTCTACTCTTGTGCAG		
		Reverse	CTCTTCCATATAGGCAGCTCTCCC		

### Sequencing and amplification analysis

**Fig 2:**
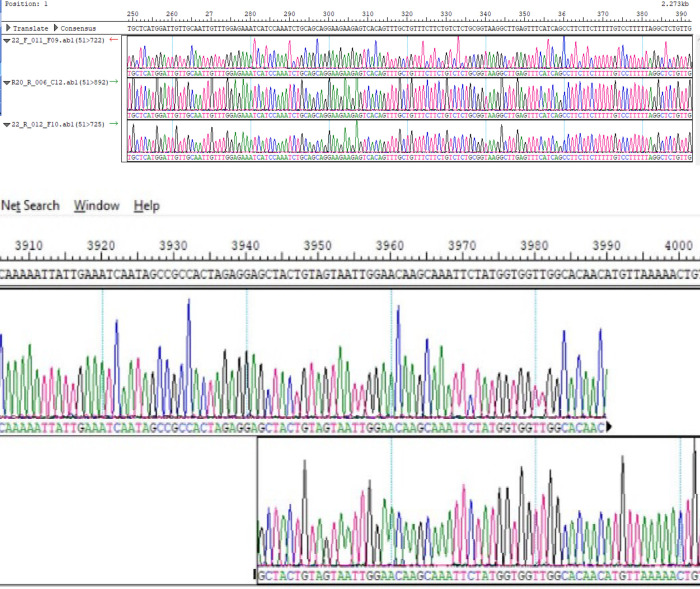
a & b: Snapshots of N-region and ORF1ab region of the assembled individual sequence chromatograms.

The PCR fragments were amplified and sequenced using the primers listed in Table 1. The gel images obtained from the gel electrophoresis of the amplicons from the PCR process using the designed primers are shown in Fig 1 below. Sequence data with read lengths ranging from 500-900bp per primer were assembled against the reference strain (NC_045512.2) into a single contiguous sequence using DNASTAR Lasergene 17 SeqMan ultra. The contig was “BLASTed” against the sequence data available in Gen-Bank. The result showed 99.97%identity with the reference sequence (NC_045512.2) and other sequences as highlighted in Table 2. Also, the amino acid identity ranges between 99.52%-100% (Table 2) depending on the region been considered. The sequence has been deposited in GenBank with accession number: MT576584.1. Other sequences generated using sets of primers from Table 1 above are: MT344135.1 and MT994707 from different samples. Fig 2 shows snapshots of areas of the assembled sequence chromatograms.

**Table 2:** Comparison of the level of identity of the sequence generated (MT576584.1) against isolates across other regions.

ACCESSION NUMBER/LOCATION	NUCLEOTIDE IDENTITY (%)	AMINO ACID IDENTITY (%)	Selected Coding Regions (%)					
			ORF1ab	ORF1ab	RdRp	S-gene	E-gene	N-gene
NC_045512.2/China	99.97	99.95	99.98	99.96	100	99.97	100	99.76
LC547526.1/Japan	99.99	99.98	99.99	99.98	100	100	100	100
MT358639.1/Germany	99.99	99.98	99.99	99.98	100	100	100	100
MT451147.1/Australia	99.98	99.96	99.99	99.98	100	100	100	100
MT470103.1/France	99.98	99.97	99.99	99.96	100	100	100	100
MT461606.1/USA	99.98	99.96	99.99	99.98	100	100	100	99.92
MT292570.1/Spain	99.97	99.93	99.98	99.94	100	99.97	100	99.68
MT457389.1/Italy	100	100	NA	NA	NA	NA	NA	100
MT350282.1/Brazil	99.95	99.89	99.96	99.94	100	99.95	100	99.76

Abbreviations: ORF = open reading frame, RdRp = RNA-dependent-RNA polymerase, S- Spike or surface glycoprotein, Envelope and N- nucleocapsid protein.

### Variation analysis

**Fig 3:**
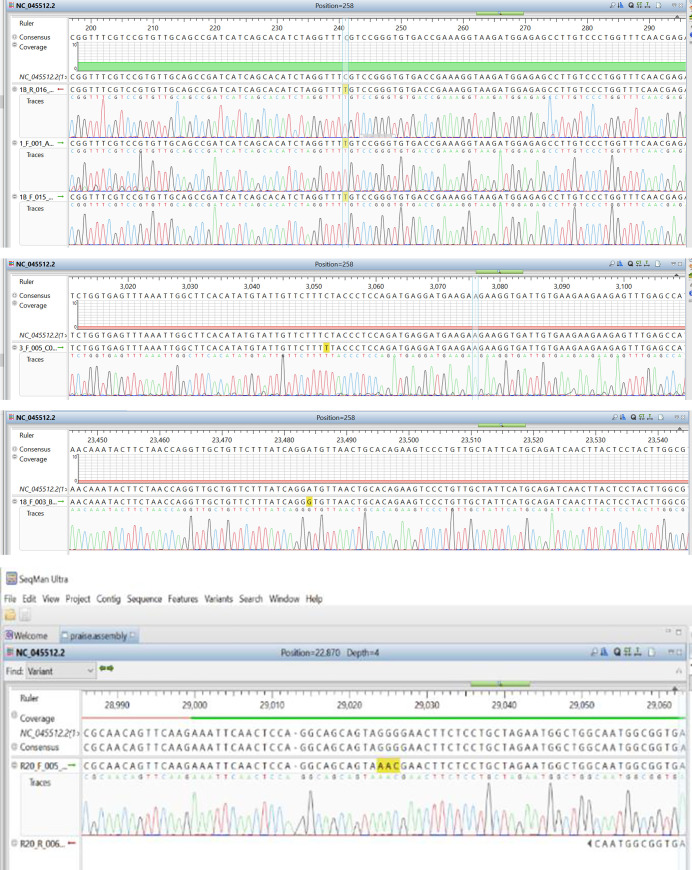
a–Nucleotide variation at position 201 (5’ NCR). b—Nucleotide variation at position 2997 (ORF1ab–region). c-Nucleotide variation at position 23363 (Spike-gene). d—Nucleotides variation at position 28841–28843 (N-region).

In comparing the assembled sequence with the reference sequence–NC_045512.2, there were notable nucleotide differences in some positions as shown in Table 3. Fig 3A–3D shows the points of variations in the assembled sequence chromatograms.

**Table 3:** Variation analysis of nucleotide bases and amino acids of the sequence (MT576584.1) as against the reference strain.

Region	Nucleotide position	Base call on consensus	Base call on reference	Amino acid/position on reference sequence	Amino acid/position on generated sequence
5’ NCR	201	T	C	-	-
ORF1a	2997	T	C	-	-
ORF1b	14368	T	C	314 P	314—L
ORF1b	16535	T	C	-	-
ORF1ab	20334	G	A	6704—I	6704 -V
S-gene	23363	G	A	614—D	614- G
N-gene	28841–28843	AAC	GGG	203–204—RG	203–204—KR

Note: “-“means no change in amino acid.

### Phylogenetic analysis

**Fig 4:**
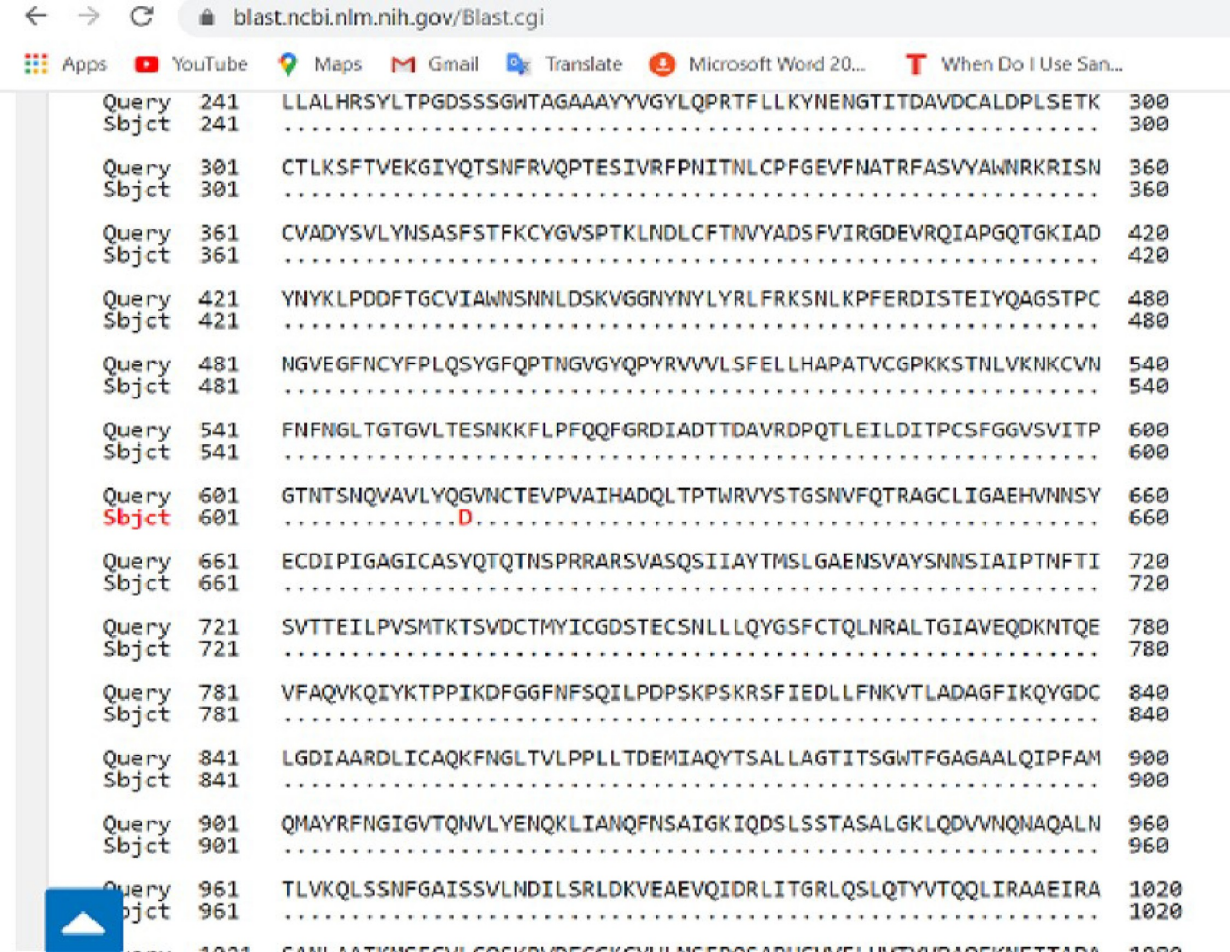
Pairwise with dots alignment of reference protein (YP_009724390.1) and analysed sequence protein (QKK12863.1) showing point (614) of protein variation.

Result from the phylogenetic analysis shows clustering around a Japanese (LC547525.1), Australian (MT450944.1) and French (MT470103.1) isolates (Fig 4).

## Discussion

Many developing countries in the world are limited in resources to set up latest sequencing platforms, however, in order to attain a highly effective tools for diagnosis and management of an infection on a global level, there is a need to have representative sequences of such infection causing organism generated from across the world. This will help to put into consideration the available strains of such pathogen circulating the globe when vaccines and drugs are been developed. Sanger sequencing platform provides a good alternative method to generate sequences by laboratories that lack NGS methods.

This study reports an identity of 99.97% between the full genome sequence generated and the reference strain at the nucleotide level and a 99.95% at amino acid level. Other sequences sampled from GenBank as shown in Table 2 had identity of 99–100% at both nucleotide and amino acid levels. The sequence analysed is not 100% identical to the reference genome, but 99.97% identity shows that there is a minimal or insignificant variation as the virus spreads across several environmental regions, from China to Nigeria at the moment. However, this seemingly insignificant variation, needs to be further studied as its corresponding changes in the protein building block could result in a mutation that will alter the behaviour of the virus. Lu and colleagues **[16]** established that coronaviruses have an average evolutionary rate of about 10^−4^ base substitution site per year with mutations arising at every replication cycle however that has not been the case with SARS-CoV-2 which has remained majorly conserved even after 6 months of its outbreak **[7]**.

Nevertheless, in this study, we have observed some variations at ORF1b, S-region and N-region leading to changes in the corresponding amino acid sequence as highlighted in Table 3 above. ORF1b which covers from 13468nt to 21555nt of the entire genome partly codes for the RdRp gene which is involved in genome replication, mRNA synthesis, or RNA recombination. It is also important for the survival of viruses and play a role in their evolution **[5].** Though there is 100% identity at the RdRp gene among our sequences, the reference and other sequences (Table 2), surveillance is needed as the virus evolves.

**Fig 5:**
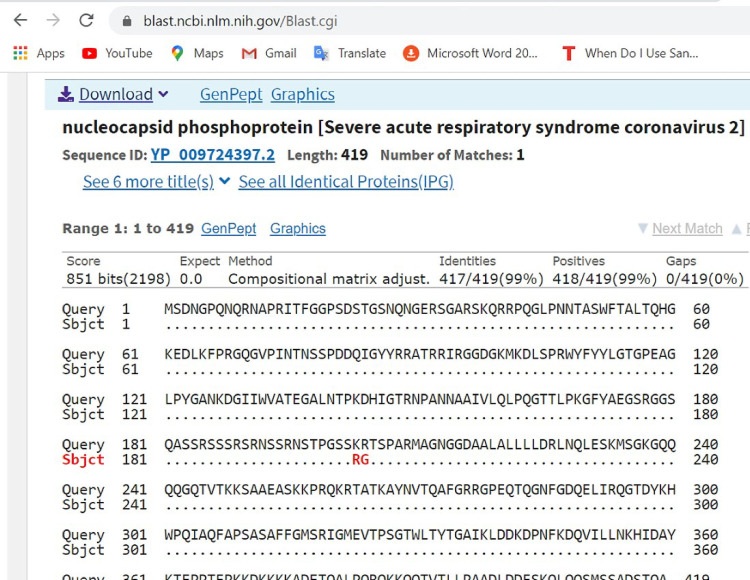
Pairwise with dots alignment of reference protein (YP 009724397.2) and analysed sequence protein (QKK12871.1) showing point (203–204) of protein variation.

The spike (S) gene is an important gene in the virus morphology and plays a major role in attachment of the viral particle to the receptor cells in its host range determination and in membrane fusion **[17].** In this study, a nucleotide variation was noticed at nt23363 which resulted in a corresponding amino acid change from D (Aspartic acid) to G (Glycine) at amino acid position 614 as seen in Fig 5 above. We are of the opinion that further studies are necessary to investigate the possible origin and effect of this variation especially with respect to the pathogenesis of the virus.

**Fig 6:**
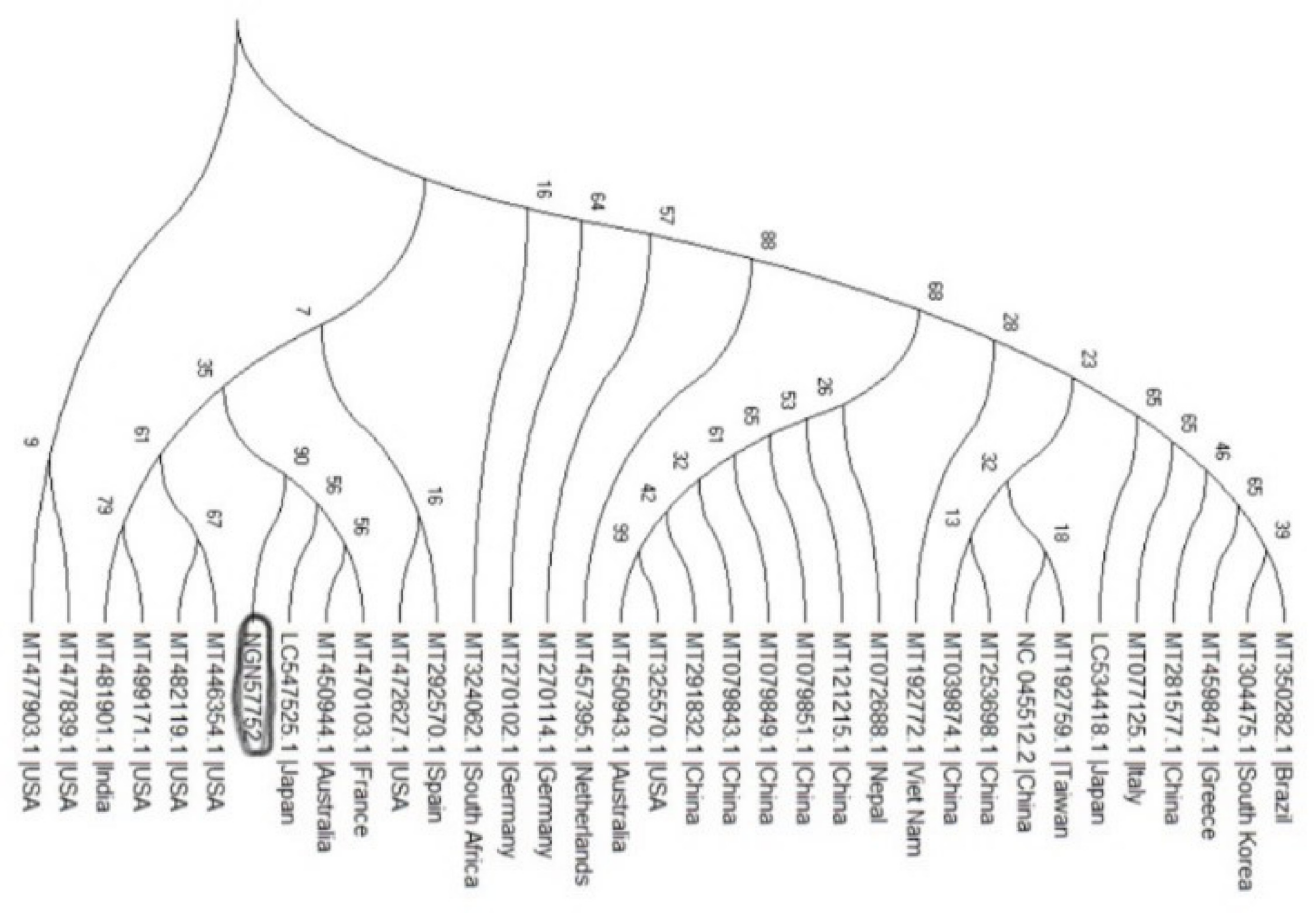
Phylogenetic tree showing the relationships of the sequences from Nigeria with others.

Furthermore, mutations at the nucleoprotein region nt28841, nt28842 and nt28843, where “AAC” replaces “GGG” of the reference strain were also observed. This led to corresponding variations in the amino acid coding, at position aa203, where K (Lysine) replaced R (Arginine) and at position aa204, where R (Arginine) replaced G (Glycine) as seen in Fig 6 above. It has been suggested that the C-terminal domain of the Nucleoprotein of coronaviruses plays an important role of binding the gRNA packaging signal leading to selective genome incorporation **[18,19]**. The nucleoprotein has also been associated with viral RNA replication and transcription; protection of the genome and possibly facilitate genome transport to the budding site **[20]**. Thus, a possible mutation at that region could play a vital role in viral fitness and even pathogenesis. Further investigation is required to elucidate the possible effect of these variations.

### Conclusion

The use of Sanger sequencing technology for successful sequencing of whole genome of SARS-CoV-2 in this study is instructive that the technology could be useful for facilities, lacking NGS but have Sanger technology, especially those in resource constrained settings. The variations in the sequence obtained, highlight the need for molecular surveillance to generate more genomic data from Africa, even as SARS-CoV-2 spreads and adapts to various environmental conditions globally.
